# Stress fiber contraction induces cell body rotation in single keratocytes

**DOI:** 10.2142/biophysico.bppb-v22.0023

**Published:** 2025-10-04

**Authors:** Chika Okimura

**Affiliations:** 1 Department of Biology, Yamaguchi University, Yamaguchi 753-8512, Japan

**Keywords:** cell migration, epidermal cell, robot model, wheels

## Abstract

Single epidermal keratocytes, which are responsible for wound repair in fish, migrate while maintaining their characteristic shape: a frontal crescent-shaped lamellipodium and a posterior rugby-ball-shaped cell body. These cells are widely used in cell migration studies, especially to examine the role of actin polymerization at the leading edge of the cell. In the posterior part of the cell, stress fibers, which are bundles of actomyosin, are aligned along the seam of the ‘rugby ball.’ The ball rotates with the stress fibers during migration. The linear contraction of stress fibers appears to drive the rotation of the cell body. This review describes a conversion mechanism from linear motion to rotation driven by stress fiber contraction and soft cell body deformation, which is not found in man-made machines. We also describe a unique research method that is able to demonstrate this machinery by creating robot models. Due to their high migration rate and ease of culturing, fish keratocytes appear to be one of the best materials for studying both single cell and collective cell migration. In this review, we will also give some recent research examples of collective migration using keratocytes.

## Significance

In a migrating fish epidermal keratocyte, the cell body rotates with the stress fibers, which consist of contractile actomyosin bundles. The linear contraction of these stress fibers causes rotation of the cell body. Conversion of linear motion into rotational motion is used in many machines, but these require precise motion of complex rigid parts. Migrating keratocytes, however, operate on a different principle. They appear to have achieved a simple conversion strategy that uses their own flexibility, a characteristic of living organisms. This unique system has future potential for application to robots.

## Introduction

**Figure 1:**
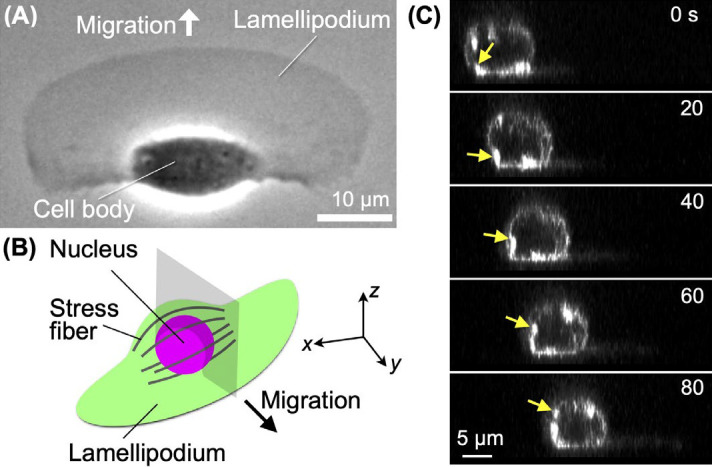
Rotation of the cell body with stress fibers in a migrating keratocyte. (A) Phase-contrast image of a typical migrating keratocyte. (B) A 3D schematic of a migrating keratocyte. Stress fibers surround the nucleus. Gray: positions of the optical sections shown in (C). (C) Stress fibers at the gray optical sections in (B), constructed from the 3D recording under confocal microscopy. F-actin (Alexa Fluor^®^ Phalloidin). The images are from Figure 4 of [12].

Crawling cell migration plays an essential role in biological phenomena, including development [1,2], immune system function [3,4], and wound healing [5,6]. The process of re-epithelialization in wound healing is achieved by the migration of surrounding epidermal cells to the wound site. In fish, epidermal keratocytes play this role in the re-epithelialization process. A single keratocyte isolated from a fish shows fast migration while maintaining its characteristic shape, comprising a frontal crescent-shaped lamellipodium and a rear rugby-ball-shaped cell body (Figure 1A) [7–12].

In 1984, Euteneuer and Schliwa made an interesting observation: lamellipodial fragments of keratocytes continued to migrate [13]. Their report suggests that the cell body can function as merely a passive cargo that is not required for migration. On the other hand, Anderson et al. (1996) subsequently observed that the translocation of the cell body continued after the arrest of lamellipodial protrusions using cytochalasin B [14], which inhibits the formation of actin filaments. Furthermore, Anderson et al. (1996) and Svitkina et al. (1997) observed, under phase-contrast microscopy, that particles within the cell bodies of keratocytes rotated along their circumference, suggesting that the cell body moves forward autonomously while rotating [14,15].

The following is currently known about the migration mechanism of many cell types. At the leading edge, actin polymerization pushes the front, and contraction of actomyosin stress fibers retract the rear. The front-to-rear alignment of the ventral stress fibers [16–18] seems to be ideal for posterior contraction. However, unlike most migrating cells, keratocyte stress fibers are positioned to connect the left and right focal adhesions perpendicular to the direction of migration (Figure 1B) [19–21]. It does not appear that the stress fibers in this alignment effectively retract the rear. Why stress fibers in keratocytes align perpendicularly to the direction of migration is therefore an interesting question.

In this review, we describe the unique rotating behavior of keratocyte cell bodies and the recently-revealed driving mechanism that uses stress fiber contraction.

## Keratocyte cell body rotation driven by stress fibers

Anderson et al. (1996) observed the particles in the cell body of a migrating keratocyte under phase-contrast microscopy [14]. They noted that particles close to the substrate moved backwards, while particles close to the top of the cell body moved forward, suggesting that the cell body itself is rotating. It is interesting to consider what drives this rotation.

They showed that not only particles in the cytoplasm but also membrane proteins in the cell cortex and the nucleus in the center of the cell body rotate, based on their observations of concanavalin A-coated fluorescent beads and autofluorescence of the nucleus. The cell body of a keratocyte is shaped like a slightly deflated rugby ball, with a flat bottom [11]. Stress fibers, which are bundles of actomyosin, are arranged along the seams of this rugby ball. We observed the rotation of these stress fibers using sequential three-dimensional (3D) recordings (Figure 1C) [11,12]. Treating keratocytes with low concentrations of blebbistatin, an inhibitor of myosin II ATPase, causes disassembly of the stress fibers [21,22]. Keratocyte cell bodies without stress fibers did not rotate [11]. Membrane proteins, nuclei, and stress fibers might thus be the power source for cell body rotation, with stress fibers being the most promising candidate.

The power source should rotate at the fastest speed. We simultaneously stained the nucleus and stress fibers, or membrane proteins and stress fibers, and recorded sequential 3D images under confocal microscopy to compare the rotational angular velocities [12]. As expected, stress fiber rotation was the fastest and is therefore the most likely to be the source of the driving force for the rotation of the whole cell body.

## Conversion from linear contraction of stress fibers to cell body rotation

Stress fibers are molecular machinery that contracts linearly, similar to myofibrils. If stress fiber contraction causes cell body rotation, some kind of linear-to-rotational conversion mechanism must be at work. Conversion of linear motion to rotation is an important mechanism in man-made machinery. For example, in an automotive cylinder engine, the linear motion of the pistons is ultimately converted into the rotational motion of the wheels. Even combustion engines, which have a history of over a century, require complex mechanisms, such as crankshafts and connecting rods, to convert linear motion into rotational motion. On the other hand, it is unlikely that cells have such sophisticated structures as crankshafts. Instead, cells are soft, unlike many man-made machines. Understanding how keratocytes convert the linear motion of stress fibers into rotational motion, therefore, is of considerable interest.

**Figure 2:**
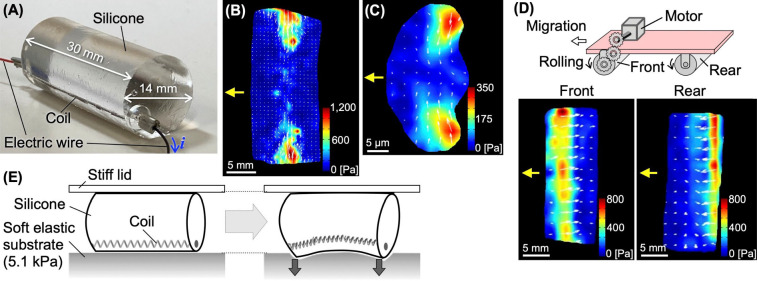
Mechanism of rotation of the cell body robot model. (A) Photo of the robot model. It consists of a soft silicone gel cylinder and a coil that contracts when an electric current (*i*) is applied. (B and C) Traction forces exerted by the robot model (B) and by a migrating keratocyte (C). Images (B and C) are from Figure S2 of [12]. (D) Schematic of the vehicle robot model (Top). Traction forces exerted by the front wheel of the vehicle robot model (Front) and the rear (Rear). The yellow arrows in (B–D) indicate the direction of migration of each robot model. (E) Schematic of the cell body robot operation on a soft substrate. Upward displacement is restricted by the lid. Before (Left) and after (Right) contraction of the coil.

To elucidate the mechanism by which stress fibers convert linear contraction into rotational motion, we constructed a robot model that mimics the cell body of a keratocyte (Figure 2A) [12]. The robot consists of a soft silicone cylinder, which resembles a cell, and a coil, which resembles a stress fiber, embedded in its side. The coil contracts when an electric current is applied to it. When the coil is made to contract, the robot rotates.

Moving objects must exert traction forces on the substrate to be able to move. The distribution of traction forces depends on the mechanism of motion of the given moving object. Comparing maps of the traction force exerted by each moving object is therefore a useful technique for comparing the principles of motion of different moving objects. If different moving objects move on a substrate using the same mechanism, their traction force distributions should be similar. The traction force map exerted by the robot model (Figure 2B) was similar to that exerted by the migrating keratocyte (Figure 2C). We constructed another robot model, consisting of a vehicle with front wheel drive and a trailing rear wheel (Figure 2D). The wheels are made of hard acrylic, not soft silicone, and their diameter and length are the same as in our robot model of a keratocyte cell body (Figure 2A). The traction force map of our cell body robot model (Figure 2B) was completely different from that of the vehicle robot with front wheel drive and a trailing rear wheel (Figure 2D). These results suggest that the rotation of both the cell body robot model and the real keratocyte cell body are driven by similar mechanisms.

**Figure 3:**
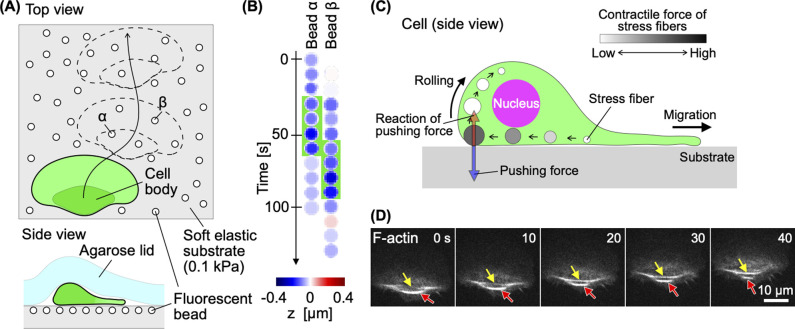
Mechanism of keratocyte cell body rotation. (A) Migration of a keratocyte on a soft substrate. The fluorescent beads were embedded in the surface of the substrate. Schematic of the experiment. (B) Sequential images of the height of the two beads α and β, over which the left and right sides of the cell body pass as illustrated in (A). The time periods when the cell body was on the site labeled with beads α and β are indicated by the green background. (C) How linear contraction of stress fibers is converted into rotation. (D) Sequential images of F-actin (Alexa-phalloidin) at the bottom of a migrating keratocyte. Yellow and red arrows indicate single stress fibers.

This similarity suggests that the mechanism of rotation of a keratocyte cell body can be predicted from that of the robot model. The silicone cylinder of the cell body robot deforms when the coil contracts, and exerts a force against the substrate at both ends. This fact was confirmed by the robot’s operation on a soft substrate (Figure 2E) [12]. Keratocytes also migrated while denting the soft substrate at both ends of their cell bodies (Figure 3A, B) [12]. The single-coil toy robot cannot reproduce the continuous migration of keratocytes. We therefore constructed another robot with three coils. By sequentially applying current pulses to the coils, the robot continuously rotated and advanced [12]. Based on these results, we predicted that the following process would be repeated in migrating keratocytes for cell body rotation: First, the ventral stress fibers thicken as they move posteriorly, thereby increasing contractility. Next, when the contractile force exceeds a threshold at the posterior end, the stress fibers detach from the substrate deforming the soft cell body. The deformed cell body then pushes against the substrate, causing itself to rotate (Figure 3C). As predicted, the stress fibers became thicker as they moved toward the posterior end (Figure 3D yellow arrow), and thinner after detaching from the substrate at the posterior end (Figure 3D red arrow).

## Role of cell body rotation in keratocyte migration

The rotational cell body resembles the wheels of a vehicle. The predicted migration velocity of keratocytes, which is the product of the radius and the angular velocity of the cell body, is only half the measured migration velocity [11,14,15]. This fact indicates that cell body rotation cannot be the main source of cell propulsion. As in other cell types, actin polymerization pushes the leading edge of the lamellipodium in keratocytes [19,23]. What, then, is the role of cell body rotation? The role of a vehicle’s wheels, other than transmitting propulsive force to the ground, is that of steering.

Cell turning to the left or right is an important function that helps cells move toward attractants and avoid dangerous extracellular environments. Cells exert traction forces on the substrate to enable crawling migration. Many migrating cells, including slow-migrating fibroblasts and fast-migrating neutrophil-like HL-60 cells and *Dictyostelium* (slime mold) cells, exert traction forces toward the cell center from their front and rear [24]. The traction forces of keratocytes, however, are mainly detected at the left and right edges, with the direction being toward the center of the cell (Figure 2C) [12,19–21,25,26]. Several investigators have reported that asymmetry in traction forces causes keratocytes to turn [23,27,28].

**Figure 4:**
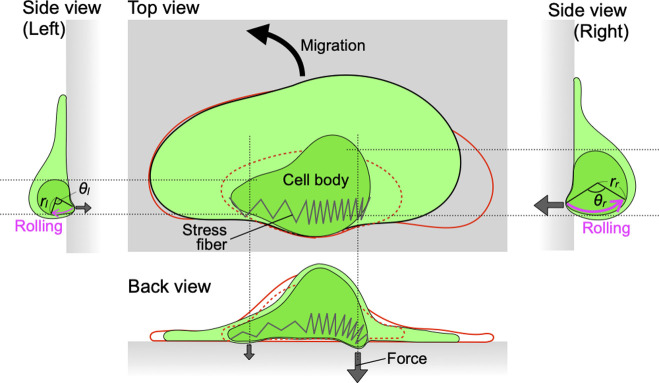
A hypothesis to describe the steering mechanism by cell body rotation resulting from stress fiber contraction. Stronger contraction of the stress fibers (right side) increases both the radius (*r_r_*>*r_l_*) and angular velocity (*θ_r_*>*θ_l_*) of the rotation, or one of them. The red and red-dotted lines show the contour of the cell and the cell body before the stress fibers contract.

Since these traction forces originate at the point where stress fibers attach to the substrate via focal adhesions [19–21,26], it is likely that the contractile force of the stress fibers is the source of the traction forces. Roy et al. disrupted stress fibers by local activation of thymosin β4, an inhibitor of actin polymerization [29]. This caused collapse of the left-right balance during crawling migration. We also showed that when a portion of the stress fibers had been ablated by laser microablation, the cell turned toward the ablated side [11,30]. The stress fibers on the ablated side would have lost their contractility. These findings suggest that asymmetry in traction forces, i.e., asymmetry in stress fiber contraction, may cause cell turning, leading us to expect that cell body rotation by stress fibers would act as a steering mechanism. First, the contraction of the stress fibers causes the focal adhesions connected to them to detach from the substrate, which deforms the cell body. The deformed cell body then pushes against the substrate, inducing its own rotation. If stress fiber contraction is asymmetric (Figure 4), the cell body will deform more on one side due to greater contraction. This increases both the radius of rotation and the force applied to the substrate, or one of them. As a result, the advancing speed of the cell increases on that side, causing the cell to turn toward the opposite side.

## Summary of stress fiber contraction-induced cell body rotation in a single keratocyte

In this review, we have shown that (I) cell body rotation in keratocytes is driven by the contraction of stress fibers that are positioned around the cell body, and (II) the underlying mechanism involves the contraction of stress fibers at the rear end of the cell floor, causing the soft cell body to deform. This deformation in turn causes the cell body to push against the substrate.

There are several organisms that use rotational mechanisms to move in space. A well-known example is the bacterial flagellar motor, which rotates the connected flagellar filament and propels the cell forward [31]. In multicellular organisms, leg joints rotate to allow the legs to be bent and extended. In the planthopper, the joints at the base of the left and right legs are linked by interlocking gears, ensuring that the left and right legs kick with equal force [32]. While a wide variety of rotational mechanisms are present in living organisms, to the best of my knowledge, no organism has been observed that utilizes the rotating mechanism itself as a wheel for locomotion, as is the case with automobiles. It would be interesting if the rotation of the cell body in a keratocyte were to function like the wheel of a car. Since actin polymerization at the leading edge is the main propulsive force for cell migration, it seems that the rotation of cell bodies does not generate propulsive force, but is most likely responsible for steering. The steering mechanism in tactic behaviors is generally considered to involve asymmetric pseudopod formation and maintenance at the cell’s leading edge in response to gradient stimuli. In fact, keratocytes exhibit galvanotaxis, and their migration direction is reversed in the presence or absence of the PI3 kinase inhibitor LY-294002, suggesting that electric fields are transduced into migration direction by two intracellular signaling pathways, including one dependent on actin polymerization [33,34]. It is unlikely that asymmetric rotation of the cell body is the sole steering mechanism. Nevertheless, proposing a novel mechanism that has not been previously considered adds value to this review. Evidence to support the hypothesis that cell body rotation is involved in steering might be obtained in the future by closely observing the rotation of stress fibers in migrating keratocytes.

## The potential of keratocytes as a resource for research on collective cell migration

**Figure 5:**
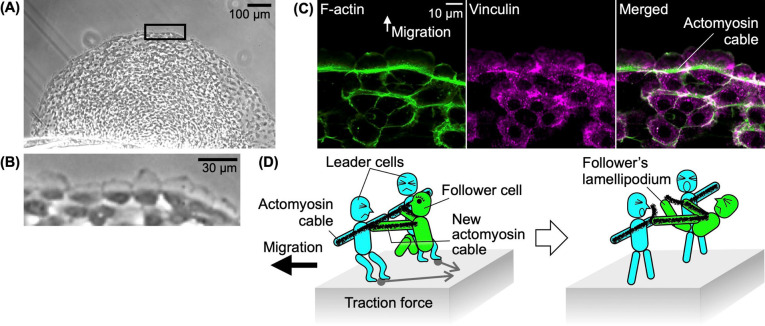
Collective migration of keratocytes. (A) A sheet-like keratocyte collective. (B) Enlarged image of the rectangle in (A). All leader cells have a single large lamellipodium. (C) Leader cells are connected to each other via actomyosin cables. F-actin (green) and vinculin (magenta). (D) An anthropomorphic illustration showing a follower cell inserting itself between two leader cells. First, the leader cells pick up a follower cell (Left). The follower then extends its lamellipodium and breaks the cable between the leaders (Right).

Single keratocytes maintain their shape while migrating, they have been used extensively in studies on the intracellular molecular dynamics of single-cell migration. However, in the fish epidermis, to repair a wound, keratocytes appear to migrate not singly (Figure 1A) but collectively (Figure 5A) toward it. Fish are poikilothermic animals, and their keratocytes can be cultured in Leibovitz’s L-15 medium, which is designed for use without additional CO_2_ in the cell culture atmosphere. Keratocytes can be therefore cultured at room temperature and without an additional CO_2_ supply. The rate of collective migration of fish keratocytes (several μm/min) [35–38] is much faster than that of epithelial cells such as human keratinocytes [39]. The faster migration of keratocyte collectives significantly reduces the time needed for experimental observation. Ease of culture and the fast migration speed of keratocyte collectives make them ideal experimental materials for the study of collective migration. All the keratocytes at the leading edge of the collective have lamellipodia, which is characteristic of leader cells (Figure 5B) [35,37]. At the same time, thick cables composed of actomyosin are distributed at the boundary of the lamellipodium and cell body of each leader cell, and are interconnected via cell-to-cell adhesion using vinculin (Figure 5C) [35,37,38]. Actomyosin cables in leader cells in keratocyte collectives appear to be transformed from stress fibers in single keratocytes through this form of connection.

We have recently found a unique mechanism in keratocyte collectives, in which cooperative interaction between two leader cells and a single follower cell can break the actomyosin cable connection between the two leader cells and admit the follower cell, which transforms into a leader cell (Figure 5D) [35]. There may still be interesting but unknown mechanisms in both single keratocytes and keratocyte collectives. We anticipate further discoveries in studies on single and collective cell migration mechanisms using fish keratocytes. Elucidating the unique mechanisms of fish keratocytes will expand our understanding of both individual and collective cell migration. It seems that there are still many fascinating biological phenomena waiting to be discovered in the organisms around us.

## Conflict of interest

The author declares no conflicts of interest.

## Author contributions

CO conceptualized the topic of this article and wrote the manuscript.

## Data availability

The data generated or analyzed during the current study are available from the corresponding author upon reasonable request.
